# The consistency of invasive and non-invasive arterial blood pressure for the assessment of dynamic cerebral autoregulation in NICU patients

**DOI:** 10.3389/fneur.2022.1032353

**Published:** 2022-12-14

**Authors:** Weijun Zhang, Hongji Lu, Jia Liu, Aihua Ou, Pandeng Zhang, Jingxin Zhong

**Affiliations:** ^1^Department of Brain Function, The Second Affiliated Hospital of Guangzhou University of Chinese Medicine, Guangzhou, China; ^2^Department of Neurological Intensive Care Unit, The Second Affiliated Hospital of Guangzhou University of Chinese Medicine, Guangzhou, China; ^3^Department of Advanced Computing and Digital Engineering, Shenzhen Institutes of Advanced Technology, Chinese Academy of Sciences, Shenzhen, China; ^4^Department of Big Data Research of TCM, The Second Affiliated Hospital of Guangzhou University of Chinese Medicine, Guangzhou, China

**Keywords:** dynamic cerebral autoregulation, invasive arterial blood pressure, non-invasive arterial blood pressure, cerebral blood flow, transfer function analysis

## Abstract

**Background:**

Studies of the clinical application of dynamic cerebral autoregulation show considerable variations, and differences in blood pressure devices may be one of the reasons for this variation. Few studies have examined the consistency of invasive and non-invasive arterial blood pressure for evaluating cerebral autoregulation. We attempted to investigate the agreement between invasive and non-invasive blood pressure methods in the assessment of dynamic cerebral autoregulation with transfer function analysis.

**Methods:**

Continuous cerebral blood flow velocity and continuous invasive and non-invasive arterial blood pressure were simultaneously recorded for 15 min. Transfer function analysis was applied to derive the phase shift, gain and coherence function at all frequency bands from the first 5, 10, and 15 min of the 15-min recordings. The consistency was assessed with Bland–Altman analysis and intraclass correlation coefficient.

**Results:**

The consistency of invasive and noninvasive blood pressure methods for the assessment of dynamic cerebral autoregulation was poor at 5 min, slightly improved at 10 min, and good at 15 min. The values of the phase shift at the low-frequency band measured by the non-invasive device were higher than those measured with invasive equipment. The coherence function values measured by the invasive technique were higher than the values derived from the non-invasive method.

**Conclusion:**

Both invasive and non-invasive arterial blood pressure methods have good agreement in evaluating dynamic cerebral autoregulation when the recording duration reaches 15 min. The phase shift values measured with non-invasive techniques are higher than those measured with invasive devices. We recommend selecting the most appropriate blood pressure device to measure cerebral autoregulation based on the disease, purpose, and design.

## Introduction

The concept and term of cerebral autoregulation (CA) were first proposed by Lassen (1) and refers to an intrinsic ability of the brain to maintain an adequate cerebral perfusion pressure (CPP) or cerebral blood flow (CBF) in the presence of arterial blood pressure (ABP) changes (2). The cerebral blood flow velocity (CBFV) can represent the CBF on the assumption of constant vessel diameter. The advent of transcranial Doppler ultrasound (TCD) (3) and the technological developments of ABP devices (4) have allowed the analysis of dynamic cerebral autoregulation (dCA), which refers to short-term CBFV responses to induced or spontaneous changes in blood pressure (BP). Currently, most research groups use spontaneous instead of induced BP fluctuations to assess dCA, and transfer function analysis (TFA) has become a popular analytical approach adopted in previous studies (5). However, the implementation of TFA involves the choice of a relatively large number of settings in practice, which may hinder progress toward clinical application. Thus, the standardization of settings warrants exploration.

CA can be impaired under physiological and pathological conditions, such as traumatic brain injury (TBI) (6), subarachnoid hemorrhage (SAH) (7), and intracerebral hemorrhage (ICH) (8), and is an independent outcome predictor in some disorders (9). Therefore, more active and individualized blood pressure management based on dCA may improve outcomes. However, studies on the clinical application of dCA show considerable variations (10), which is difficult to reference in other studies. Despite a consensus white paper from the Cerebral Autoregulation Research Network (CARNet-www.car-net.org) improving the standardization of parameters and settings adopted for TFA applications in studies of dCA (5), different blood pressure instruments were used in previous studies, which may be one of the reasons for the considerable variations.

Four of the most common methods for ABP measurements include arterial line measurement, finger photoplethysmography, sphygmomanometry, and tonometry (11). The first two methods allow for the continuous monitoring of ABP. The arterial line is an invasive blood pressure (IBP) measurement method, while finger photoplethysmography is a non-invasive blood pressure (NIBP) measurement technique (11). When evaluating dCA, one of two methods is chosen. Invasive techniques are normally regarded as the “gold standard” but can only be used under certain conditions (12), such as in some critically ill patients. In contrast, non-invasive methods are much more widely used (4). Since NIBP can adequately assess blood pressure variability (13), the biases are relatively small over a wide range of autoregulation metrics (12). As methods for assessing dCA are translated from the research laboratory to clinical practice, establishing any differences in results that could be ascribed to the techniques adopted to record continuous ABP becomes increasingly important (14). However, few studies have examined the consistency between invasive and non-invasive arterial blood pressure measurement techniques for the assessment of dCA. Therefore, we attempt to investigate how these two BP methods affect the parameters of the dCA. In addition, based on the effect of data length on TFA parameters in clinical practice (15), we also observed whether the consistency was affected by the recording duration.

## Methods

### Subjects

The study was approved by the Ethics Committee of the Second Affiliated Hospital of Guangzhou University of Chinese Medicine (ZE2019-247-01). Patients admitted to the neurological intensive care unit (NICU) at the Second Affiliated Hospital of Guangzhou University of Chinese Medicine from October 2020 to December 2020 were eligible for the study. A radial artery catheter was placed in all subjects to monitor ABP for clinical purposes. Patients with bilateral poor temporal windows found in TCD, middle cerebral artery (MCA) peak flow velocity >300 or < 40 cm/s, atrial fibrillation found in electrocardiography, poor non-invasive ABP signal or poor cooperation were excluded at initial screening.

### DCA measurement

Measurements were performed in the NICU room by the same professional technician in the morning. The room was a temperature-controlled environment of 22–24°C. All subjects received breathing assistance from a ventilator (Evita V300, Drager, Germany) and were fed liquid nutrition through a small, lightweight, portable, and accurate enteral feeding pump (Flocare, Nutricia, Netherlands). Data were collected when patients were in a supine position. CBFV was assessed using TCD (EMS-9PB, Delica, China). Bilateral MCAs were monitored at a depth of 45–60 mm through temporal windows with 2-MHz probes attached to a head frame. BP was measured simultaneously by the bedside monitor (BSM-6501C, Nihon Kohden, Japan) from the intravascular catheter inserted into the left radial artery and by servo-controlled finger photoplethysmography (FMS-8C, Delica, China). A finger cuff of appropriate size was placed on the left middle finger (4), and the position of the hand was always at heart level. The finger cuff was repositioned until a stable waveform was achieved with the servo-adjust on, and waveforms were considered stable after 5 min had passed and the interval between the “physical” procedure exceeded 30 beats (16). After the recording was stable, the “physical” procedure, an intermittently occurring calibration routine, was turned off. Continuous CBFV and continuous invasive and non-invasive ABP were recorded simultaneously for each subject for 15 min. All recordings were required to be of good quality, showing a reduced presence of noise and absence of artifacts with the clear visualization of each waveform. All analog signals were digitized and stored for editing and offline analysis. The data sampling frequency was 125 Hz.

### Data analysis

The data were processed using MATLAB (MathWorks, USA). The raw data, which were not removed or interpolated, were used for analysis. The invasive ABP and non-invasive ABP were synchronized with CBFV on the signal to eliminate the time delay. The dynamic relationship between ABP and CBFV was analyzed by TFA based on the algorithm provided by CARNet. It can be calculated as follows:


$$\begin{array}{l} \;H\left(f\right)=\frac{Sxy\left(f\right)}{Sxx\left(f\right)}\\ \;\Phi \left(f\right)=\tan^{-1}\;\left[\frac{H_{I}\left(f\right)}{H_{R}\left(f\right)}\right]\\ |H\left(f\right)|=\sqrt{|H_{I}\left(f\right){|}^{2}+|H_{R}\left(f\right){|}^{2}}\\ \gamma^{2}\left(f\right)=\frac{|Sxy\left(f\right){|}^{2}}{Sxx\left(f\right)Syy\left(f\right)} \end{array}$$

where Φ(*f*), |*H*(*f*)|, and γ^2^(*f*) represent the phase shift, the gain, and the magnitude-squared coherence function, respectively; *H*(*f*), *H*_*I*_(*f*), and *H*_*R*_(*f*) represent the transfer function, the imaginary component of the transfer function, and the real component of the transfer function, respectively; and *Sxx*(*f*), *Syy*(*f*), and *Sxy*(*f*) denote the averaged autospectra of ABP, the averaged autospectra of CBFV and the cross-spectra of ABP and CBFV, respectively. In this algorithm, the anti-leakage window was a Hanning window, whose length was 90 s with 50% superposition. The 5-, 10-, and 15-min recordings comprised 5, 12, and 19 windows of data segments, respectively. Then, the TFA calculated the phase shift, gain, and coherence function between the ABP and CBFV at the very low frequency (VLF, 0.02–0.07 Hz), low frequency (LF, 0.07–0.20 Hz), and high frequency (HF, 0.20–0.50 Hz) bands. The phase shift in the LF band or gain in the VLF band reflects the CA level, and the coherence function reflects the linear correlation between CBFV and BP.

In order to increase the reach of this work, time domain indices, such as Mx_a, Sx_a, were also calculated using software ICM+ invented by Brain Physics Laboratory of Cambridge University.

### Statistical analysis

The current study sample size conforms to the rule of thumb for sample size of a study (17). Statistical data were analyzed using SPSS Statistics 17.0 (SPSS Inc., Chicago, USA). The normality was checked using the Shapiro–Wilk test. Normally distributed data are expressed as the mean ± standard deviation (SD), and non-normally distributed data are expressed as the median with interquartile range (IQR). The agreement between dCA parameters derived from invasive and non-invasive ABP devices was assessed with Bland–Altman analysis (18). The differences between the dCA parameters from the FMS-9C and catheter for each subject were plotted against the mean of these two parameters. Assuming that the differences are normally distributed (Gaussian), 95% of differences will lie between the mean difference ± 1.96 standard deviation of the differences. A small mean difference indicates small intermethod bias, while a small variance indicates good intermethod agreement. The consistency was evaluated according to the number of points outside the 95% confidence interval and the maximum difference within the 95% confidence interval, as well as the clinical acceptability. For an additional assessment of consistency, the intraclass correlation coefficient (ICC) (19) was used. A two-way random, absolute agreement model was chosen for ICC estimates. ICC values < 0.40 indicate poor reliability, those more than 0.75 indicate good reliability, and those more than 0.90 indicate excellent reliability. To analyze intermethod differences, a paired-samples *t*-test or Wilcoxon signed-rank test was used. *P-*values < 0.05 were considered statistically significant.

## Results

Six patients (63.0 ± 11.0 years, 3 males) were finally included in the study. Admission diagnoses included acute intracerebral hemorrhage, acute ischemic stroke, sequelae of cerebral infarction, endovascular therapy for basilar artery occlusion, and endovascular therapy for right middle cerebral artery stenosis. The characteristics of the patients are listed in Table 1. The mean IBP, NIBP and CBFV in the first 5, 10, and 15 min of the 15-min recordings are provided in Table 2. The dCA parameters (phase shift, gain, and coherence function) in all frequency bands are given in Table 3.

**Table 1:** Baseline characteristics of the patients.

**Parameters**	**Study population *n* = 6**
Age, years (mean ± SD)	63.0 ± 11.0
Gender sex (male/female)	3 / 3
**Diagnosis, n (%)**	
Acute intracerebral hemorrhage	2 (33)
Acute ischemic stroke	1 (17)
Sequelae of cerebral infarction	1 (17)
After endovascular therapy	2 (33)
Comorbidities, *n* (%)	
Hypertension (HTN)	5 (83)
Diabetes mellitus (DM)	3 (50)
Chronic kidney disease (CKD)	0 (0)
Peripheral arterial disease (PAD)	1 (17)

**Table 2:** Mean invasive arterial blood pressure, mean non-invasive arterial blood pressure and mean cerebral blood flow velocity measures from the first 5, 10, and 15 min of the 15-min recordings.

**Recordings, *n* = 6**	**5 min**	***P***	**10 min**	***P***	**15 min**	***P***
IBP, mmHg	82.07 ± 14.40	0.119	82.48 ± 14.86	0.252	82.98 ± 14.96	0.500
NIBP, mmHg	94.77 ± 26.80		91.23 ± 27.41		88.13 ± 27.34	
Left CBFV, cm/sec	54.91 ± 23.58		54.98 ± 23.34		54.65 ± 23.33	
Right CBFV, cm/sec	56.07 ± 20.51		57.05 ± 20.82		57.64 ± 21.58	

**Table 3:** Baseline transfer function, Mx_a and Sx_a estimates from the **first** 5, 10, and 15 min of the 15-min recordings.

**Hemispheres**, ***n*** = **12**			**5 min**	***ICC***	***P***	***t***	***P***	**10 min**	***ICC***	***P***	***t***	***P***	**15 min**	***ICC***	***P***	***t***	***P***
Phase shift (degree)	VLF	IBP	83.23 ± 45.76	0.857	**0.001**	2.209	**0.049**	81.83 ± 38.29	0.791	**0.001**	2.120	0.058	81.32 ± 31.17	0.768	**0.001**	1.558	0.148
		NIBP	96.12 ± 39.43					94.66 ± 33.52					90.19 ± 29.30				
	**LF**	IBP	32.01 ± 23.66	0.307	0.148	1.181	0.262	34.94 ± 22.05	0.750	**0.001**	4.745	**0.001**	34.35 ± 20.83	0.909	**0.001**	2.718	**0.020**
		NIBP	42.07 ± 26.62					51.64 ± 30.08					39.54 ± 16.89				
	HF	IBP	−0.79 ± 6.97	−0.068	0.655	3.091	**0.010**	0.74 ± 7.61	0.163	0.310	0.239	0.815	0.02 ± 8.16	0.134	0.300	1.968	0.075
		NIBP	−41.57 ± 42.56					−2.18 ± 45.21					−16.60 ± 30.87				
Gain (cm/s/mmHg)	**VLF**	IBP	0.63 ± 0.13	0.169	0.280	1.367	0.199	0.61 ± 0.11	0.281	0.153	1.629	0.132	0.63 ± 0.15	0.613	**0.010**	1.469	0.170
		NIBP	0.56 ± 0.14					0.54 ± 0.11					0.58 ± 0.14				
	LF	IBP	0.83 ± 0.13	0.408	**0.007**	4.839	**0.001**	0.80 ± 0.09	0.384	**0.021**	3.820	**0.003**	0.79 ± 0.12	0.581	**0.008**	2.334	**0.040**
		NIBP	0.66 ± 0.16					0.65 ± 0.18					0.72 ± 0.16				
	HF	IBP	0.77 ± 0.11	0.463	0.065	0.020	0.984	0.75 ± 0.14	0.685	**0.006**	0.391	0.703	0.75 ± 0.15	0.796	**0.001**	0.046	0.964
		NIBP	0.77 ± 0.24					0.78 ± 0.30					0.76 ± 0.23				
Coherence Function	VLF	IBP	0.64 ± 0.11	0.865	**0.001**	3.890	**0.003**	0.64 ± 0.10	0.871	**0.001**	2.987	**0.012**	0.65 ± 0.11	0.937	**0.001**	4.320	**0.001**
		NIBP	0.60 ± 0.10					0.61 ± 0.09					0.62 ± 0.10				
	LF	IBP	0.51 ± 0.19	0.924	**0.001**	3.993	**0.002**	0.47 ± 0.22	0.920	**0.001**	3.150	**0.009**	0.46 ± 0.23	0.915	**0.001**	3.353	**0.006**
		NIBP	0.45 ± 0.19					0.41 ± 0.21					0.39 ± 0.23				
	HF	IBP	0.63 ± 0.22	0.964	**0.001**	4.031	**0.002**	0.57 ± 0.23	0.869	**0.001**	2.223	**0.048**	0.57 ± 0.25	0.666	**0.003**	1.939	0.079
		NIBP	0.58 ± 0.23					0.51 ± 0.23					0.46 ± 0.27				
Mx_a		IBP	0.19 ± 0.39	0.469	0.061	0.361	0.725	0.18 ± 0.28	0.492	**0.022**	2.250	**0.046**	0.18 ± 0.21	0.492	**0.036**	1.469	0.170
		NIBP	0.23 ± 0.32					0.03 ± 0.17					0.10 ± 0.16				
Sx_a		IBP	0.01 ± 0.35	0.503	**0.020**	2.257	**0.045**	−0.04 ± 0.20	0.543	**0.032**	0.509	0.621	−0.02 ± 0.15	0.350	0.130	0.579	0.574
		NIBP	0.20 ± 0.27					−0.01 ± 0.21					0.01 ± 0.18				

Bold values indicates P-values < 0.05.

**Figure 1:**
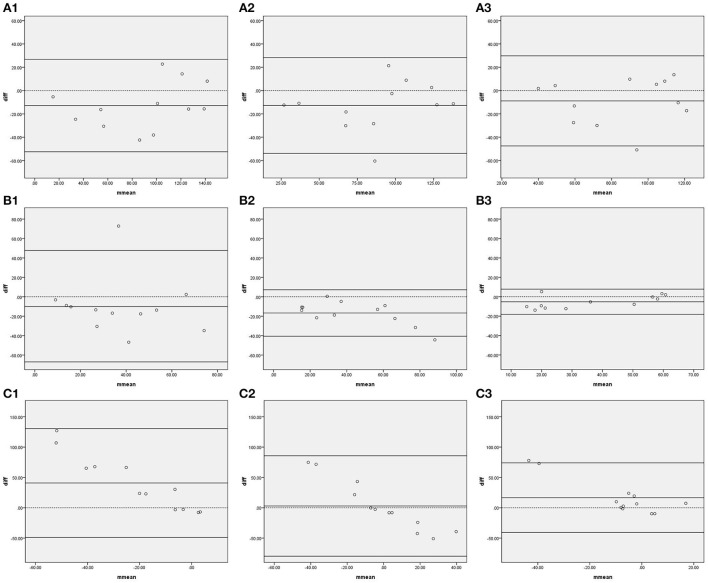
Bland—Altman plots of phase shift. In the upper left corner **(A)** represents VLF. **(B)** represents LF. **(C)** represents HF. 1 represents 5 min, 2 represents 10 min, and 3 represents 15 min; for example, **(A1)** represents the VLF band in the first 5 min. The upper and lower solid lines represent the 95% confidence intervals, the middle solid line represents the bias, and the dashed line represents the difference of 0.

Figure 1 shows the Bland–Altman plots comparing phase shifts in all frequency bands measured by IBP and NIBP in the first 5, 10, and 15 min of the 15-min recordings. In the LF band, 8.3% (1/12), 8.3% (1/12), and 0% of points were outside the 95% confidence interval for the first 5, 10, and 15 min, respectively (Figures 1B, B1–3), and the maximum differences within the 95% confidence interval were 46.77, 31.63, and 13.81, respectively; furthermore, the respective intraclass correlation coefficients were 0.307 (*P* = 0.148), 0.750 (*P* < 0.001), and 0.909 (*P* < 0.001) (Table 3). The phase shift in the LF band in the first 5-min recording did not significantly differ between the two arterial blood pressure methods, while it was significantly different in the first 10-min and 15-min recordings (Table 3).

**Figure 2:**
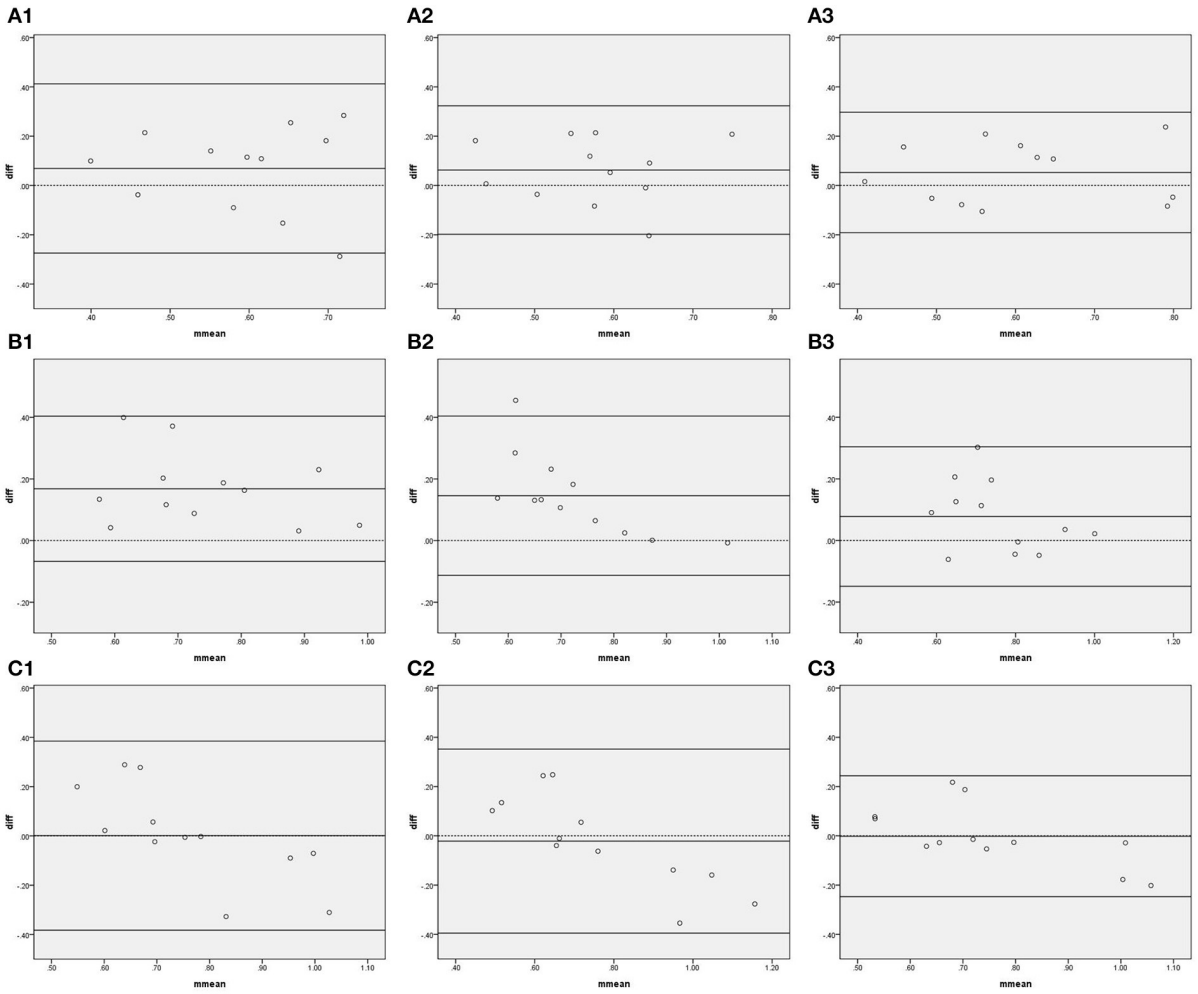
Bland—Altman plots of gain. In the upper left corner **(A)** represents VLF. **(B)** represents LF. **(C)** represents HF, 1 represents 5 min, 2 represents 10 min, and 3 represents 15 min; for example, **(A1)** represents the VLF band in the first 5 min. The upper and lower solid lines represent the 95% confidence intervals, the middle solid line represents the bias, and the dashed line represents the difference of 0.

Figure 2 shows the Bland–Altman plots comparing gain in all frequency bands measured by IBP and NIBP in the first 5, 10, and 15 min of the 15-min recordings. In the VLF band, 8.3% (1/12), 8.3% (1/12), and 0% of points were outside the 95% confidence interval for the first 5, 10, and 15 min, respectively (Figure 2A, A1–3), and the maximum differences within the 95% confidence interval were 0.28, 0.21, and 0.24, respectively; furthermore, the respective intraclass correlation coefficients were 0.169 (*P* = 0.280), 0.281 (*P* = 0.153), and 0.613 (*P* = 0.010) (Table 3). The gain in the VLF band did not significantly differ between the two arterial blood pressure methods in the first 5-, 10-, and 15-min recordings (Table 3).

**Figure 3:**
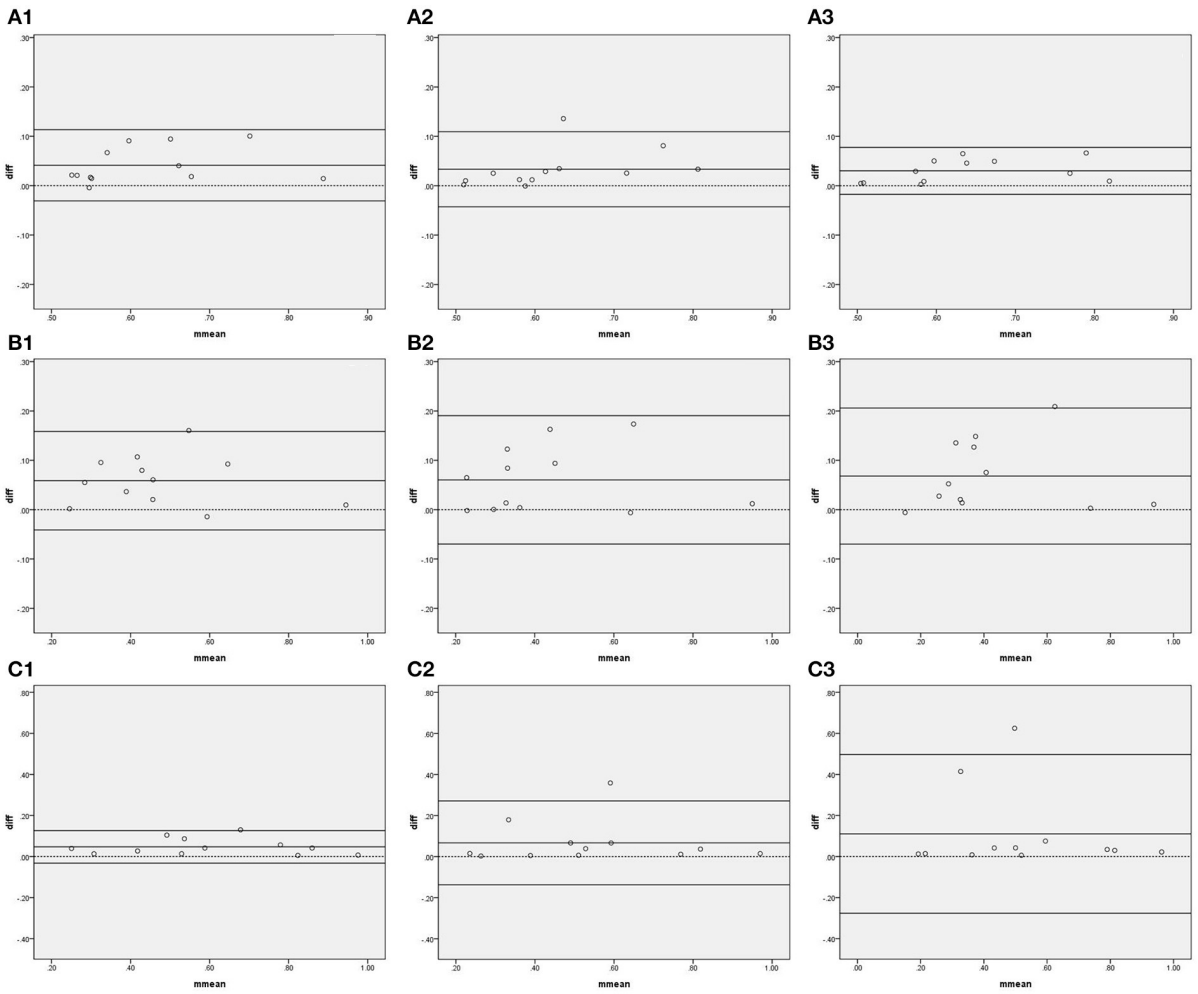
Bland–Altman plots of coherence function. In the upper left corner **(A)** represents VLF. **(B)** represents LF. **(C)** represents HF, 1 represents 5 min, 2 represents 10 min, and 3 represents 15 min; for example, **(A1)** represents the VLF band in the first 5 min. The upper and lower solid lines represent the 95% confidence intervals, the middle solid line represents the bias, and the dashed line represents the difference of 0.

Figure 3 and Table 3 show the agreement and reliability of coherence function measured by IBP and NIBP in the first 5, 10, and 15 min of the 15-min recordings. Except for the fact that the coherence function in the HF band did not significantly differ between the two arterial blood pressure methods in the first 15-min recording, the coherence function of IBP was higher than that of NIBP in all frequency bands in the first 5-, 10-, and 15-min recordings (Table 3).

**Figure 4:**
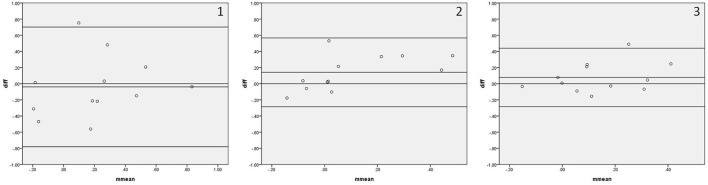
Bland—Altman plots of Mx_a. In the upper right corner, **(1)** represents 5 min. **(2)** represents 10 min, and **(3)** represents 15 min; for example, 1 represents Mx_a in the first 5 min. The upper and lower solid lines represent the 95% confidence intervals, the middle solid line represents the bias, and the dashed line represents the difference of 0.

Figure 4 shows the Bland–Altman plots comparing Mx_a measured by IBP and NIBP in the first 5, 10, and 15 min of the 15-min recordings. 8.3% (1/12), 0%, and 8.3% (1/12) of points were outside the 95% confidence interval for the first 5, 10, and 15 min, respectively (Figure 4, 1–3), and the maximum differences within the 95% confidence interval were 0.56, 0.53, and 0.25, respectively; furthermore, the respective intraclass correlation coefficients were 0.469 (*P* = 0.061), 0.492 (*P* = 0.022), and 0.492 (*P* = 0.036) (Table 3). The Mx_a in the first 5-min and 15-min recordings did not significantly differ between the two arterial blood pressure methods, while it was significantly different in the first 10-min recording (Table 3).

**Figure 5:**
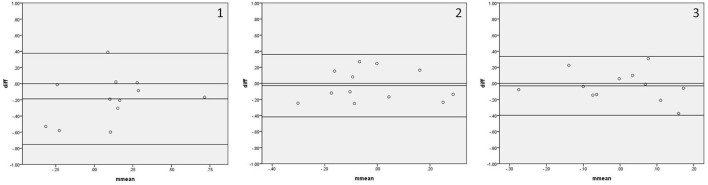
Bland—Altman plots of Sx_a. In the upper right corner, **(1)** represents 5 min. **(2)** represents 10 min, and **(3)** represents 15 min; for example, 1 represents Sx_a in the first 5 min. The upper and lower solid lines represent the 95% confidence intervals, the middle solid line represents the bias, and the dashed line represents the difference of 0.

Figure 5 shows the Bland–Altman plots comparing Sx_a measured by IBP and NIBP in the first 5, 10, and 15 min of the 15-min recordings. 8.3% (1/12), 0%, and 0% of points were outside the 95% confidence interval for the first 5, 10, and 15 min, respectively (Figure 5, 1–3), and the maximum differences within the 95% confidence interval were 0.60, 0.27, and 0.37, respectively; furthermore, the respective intraclass correlation coefficients were 0.503 (*P* = 0.020), 0.543 (*P* = 0.032), and 0.350 (*P* = 0.130) (Table 3). The Sx_a in the first 10-min and 15-min recordings did not significantly differ between the two arterial blood pressure methods, while it was significantly different in the first 5-min recording (Table 3).

## Discussion

In the current study, the consistency of IBP and NIBP for the assessment of dCA was poor at 5 min, slightly improved at 10 min, and good at 15 min. When the recording duration reached 15 min, the LF phase shift values measured by NIBP were higher than values derived from IBP, while VLF gain values did not significantly differ between the two arterial BP methods. In addition, the coherence function values measured by IBP were higher than those measured by NIBP.

Very little is known about whether different measurements of ABP may affect the calculation of dCA parameters. Lavinio et al. (20) investigated the agreement between non-invasive Mx (nMx) with the use of a finger photoplethysmograph and Mx with the use of an arterial line positioned in the radial artery, and their results showed that the non-invasive index of autoregulation nMx correlates with Mx and is sufficiently sensitive to detect autoregulation asymmetry. Sammons et al. (14) compared estimates of dCA derived from NIBP with those from invasive recordings in the aorta and found a good level of agreement between them, although significant biases were also identified. Panerai et al. (21) found that continuous estimates of dCA obtained from non-invasive measurements of beat-to-beat BP in the finger are not substantially different from corresponding values derived from intra-aortic BP recordings. Petersen et al. compared the parameters of dCA calculated using both invasive and non-invasive techniques and found that both methods yielded similar results (22). Our result is similar to those of previous studies overall, with the only difference being that a sufficiently long recording duration yielded high consistency between measurement methods. Because only the ABP methods were different, the aforementioned difference may be due to the fact that BP fluctuation requires a long time to be consistent between the two methods. Second, when the recording duration is short, the reduction in the window number may increase the variability of estimates (21). In addition, physiological variability or non-stationarity in critically ill patients is likely to be the main reason for the poor reproducibility of dCA parameters (23). Finally, differences in the study subjects may also be responsible for this difference.

Although the two methods were consistent, the intermethod differences in values warrants examination. For example, our results show that the differences in phase shift were on average 5.19 degrees lower with IBP than with NIBP. Although the details of the waveform are scarce, the location of acquisition for the BP signal may have affected the phase shift, as distal locations have a greater influence on the rhythmic changes in arteriolar tone, resulting in an increased phase shift. Alternatively, NIBP may have significantly overestimated intra-arterial systolic MF (0.07–0.14 Hz) and LF (0.025–0.07 Hz) powers (13), which may cause the difference in phase shift values. Thus, if a patient needs longitudinal assessments but NIBP or IBP methods are used at different times, the difference between methods should be recognized. For example, the dCA may be first evaluated with IBP in a critically ill patient, whereas during a later follow-up it may be evaluated with NIBP. This difference in measurement method may cause the dCA to appear to be improved, when in fact the improvement is attributable to intermethod bias. Interestingly, we have assessed the dCA in a patient with reversible leukoencephalopathy caused by rodenticide poisoning due to bromadiolone and fluroacetamide. His first dCA assessment was performed with IBP in the NICU, and result showed that the left and right phase shift in the LF band was 4.13 and 2.56 degree, respectively. 1 month later, the follow-up dCA assessment was performed with NIBP, and result showed that the left and right phase shift in the LF band was 13.80 and 6.51 degree, respectively. It seems that the dCA was improved, but considering the difference in measurement method, the dCA may be the same as 1 month before. The impaired function may need a long time to recover.

Except for the possibility of overestimating the phase shift, NIBP has lower coherence function values than IBP, perhaps because more extraneous noise is presented in the NIBP, leading to a poor signal-to-noise ratio (24). According to the calculated cutoff values for the coherence function with the recommended settings for TFA estimation provided by consensus white paper (5), dCA measured with IBP can have a more acceptable coherence function, while dCA measured with NIBP may exclude more data. More studies and discussions are needed to determine whether the cutoff values of the coherence function should be specific to the BP measurement method.

Of course, the aforementioned intermethod bias does not mean that we should preferentially use IBP to measure dCA. The advantages and disadvantages of both methods should be known. For critically ill patients, IBP should be the preferred method for dCA assessment (22). However, some contraindications exist for IBP (25), such as local infections, coagulopathy, Raynaud syndrome, Buerger's disease or surgical considerations (26). Moreover, the main complication of IBP is the temporary occlusion of the artery, while serious ischemic damage leading to necrosis and the amputation of fingers or the whole hand is also observed on rare occasions (27). Although NIBP and IBP devices are not interchangeable due to differences in accuracy and precision (28), NIBP is more widely applicable. Note that obtaining a valid waveform may be difficult in cases of severe vasoconstriction, peripheral vascular disease, or distorted fingers (29). In addition, factors such as appropriate cuff size, stabilized position, and relatively constant arm temperature, which are important to the quality of the recording, need to be ensured (30).

It is well-known that the first stage of TFA is to obtain mean values of BP and CBFV for each cardiac cycle in the time-domain. Thus, it is necessary to discuss the correlation of BP readings between the two modalities. Tanioku et al. compared BP measurements during induction of anesthesia for cardiovascular surgery, and their results showed that non-invasive mean arterial pressure (MAP) could be considered as an alternative for radial artery blood pressure (31). The agreement of non-invasive MAP was also acceptable during carotid endarterectomy (32), as well as in unselected medical ICU patients under routine clinical conditions (33). The results of other two studies indicated that the accuracy and precision of non-invasive arterial pressure measurements was reasonable for MAP and diastolic arterial pressure (34, 35). In addition, in patients undergoing major intra-abdominal surgery, the results of systolic, diastolic and mean arterial pressure measured using non-invasive device were well within the limits established for the validation of automatic arterial pressure monitoring (36). For cardiovascular postsurgical intensive care patients, Ilies et al. (37) suggested that MAP should be preferred for clinical decision making.

This study has several limitations. First, few suitable participants underwent IBP monitoring for clinical purposes, and the size of the sample was consequently small. Second, we only focused on NICU patients suffering from neurological diseases, and the applicability of these results to other disease settings is unclear. Third, whether a recording duration longer than 15 min would lead to a different result is also unclear.

## Conclusion

In summary, we found that IBP and NIBP measurements in the low frequency band were consistent for dCA assessment as the recording duration reached 15 min. The difference in TFA parameter values between methods should be considered when using different BP devices for dCA assessment. Selecting the optimal BP method for each patient to evaluate dCA may be helpful for the clinical promotion and application of dCA assessment.

## Data availability statement

The raw data supporting the conclusions of this article will be made available by the authors, without undue reservation.

## Ethics statement

The studies involving human participants were reviewed and approved by the Ethics Committee of the Second Affiliated Hospital of Guangzhou University of Chinese Medicine. The patients/participants provided their written informed consent to participate in this study.

## Author contributions

Original draft preparation and data collection: WZ. Writing-review and editing and investigation: HL. Methodology and validation: JL. Statistical analysis: AO. Data analysis: PZ. Conceptualization: JZ. All authors contributed to the article and approved the submitted version.
